# Zytokin profiles in familial Mediterranean fever patients: IL-18 as a major cytokine and potential biomarker or target

**DOI:** 10.1186/1546-0096-13-S1-P86

**Published:** 2015-09-28

**Authors:** A Igney-Oertel, J Henes, R Klein

**Affiliations:** 1 University Tuebingen, Medical Clinic, Hematology, Rheumatology, Tuebingen, Germany

## Introduction

Familial Mediterranean fever (FMF) is an autoinflammatory disease which is characterized by recurrent episodes of typical attacks with fever and abdominal pain. The pathophysiology shows an ineffective pyrin which leads to brakeless inflammation. It is not exactly known which factors lead to initiation of the attacks. Furthermore there seems to be sustained inflammation between the attacks.

IL-18 is acytokinethat belongs to theIL-1superfamily and is produced bymacrophagesand other cells. It plays an important role in the initiation and maturation of the inflammasome and is therefore able to induce severeinflammatory reactions.

## Objectives

In our study we aimed to clarify the cytokine profiles of patients with genetically confirmed FMF in mainly attack free periods and compare it with healthy controls.

We evaluated type 1 cytokines: IFN-g, TNF-b, type 2 cytokines: IL-5, IL-10, IL-13 and other inflammatory cytokines: IL-1, IL-6, IL-17, IL-18, TNF-a and GMCSF.

## Patients and methods

We examined 42 samples of 36 FMF patients between 07/2008 and 03/2015. Six patients were evaluated at two time-points. Ten healthy subjects were examined as control group. The levels of the cytokines were determined by commercial ELISA kits.

## Results

The mean age of the FMF patients was 34,1 years, 43 % with homozygotic and 57 % with combined heterozygotic mutations.

Serum levels of IL-18 (normal range up to 400pg/ml) were 1946 pg/ml in mean for FMF patients. None of the controls showed an increased level. Serum levels of homozygotic patients were with 3654 pg/ml (mean value) higher than in patients with combined heterozygotic mutations (1290 pg/ml).

Patients with a complicated form of FMF (no colchicine response, therapy with IL-1 antibody) have the highest values of IL-18 (4233 pg/ml mean). It remains unclear if this is a consequence of IL-1 antibody treatment or active disease. All of the eight FMF patients which showed no increase in IL-18, had only a mild disease-activity and heterozygotic mutations.

All other cytokines showed no significant differences between the FMF patients and the control group with a tendency of higher IL-10 and IL-13 levels. IL-1 and IL-6 levels were not elevated. The results of the 6 patients which were evaluated at two time-points showed repetitive and comparable results.

## Conclusion

Our results suggest a major role of Interleukin 18 in pathogenesis and disease course of familial Mediterranean fever. Further studies need to clarify the specific role of IL-18 and the option as a potential marker for severe disease and a target for therapy in FMF patients.

